# Immunological memory: resting on two shoulders at antigen entrance sites?

**DOI:** 10.1038/s41423-019-0263-8

**Published:** 2019-07-19

**Authors:** Clemens Wülfing, Stephan Henne, Jasmin Oehlmann, Hauke Simon Günther

**Affiliations:** Group for Interdisciplinary Neurobiology and Immunology, Biozentrum Grindel, University of Hamburg, Hamburg, Germany

**Keywords:** Neuroimmunology, Immunological memory

Memory is a term used in two important systems: the nervous system and the immune system. Both systems need to recognize alterations and threats in an ever-changing environment. As a consequence, both systems during their evolution perfected their abilities to recall earlier challenges in mounting protective responses. This ability gives both systems the power to facilitate these protective responses if confronted with the same or similar challenges again.^1–3^ In the field of psychoneuroimmunology, studies over the past decades have shown that the nervous system and the immune system also share many other features and that they interact in a form of bidirectional communication. Evidence has been reported for afferent like and also efferent innervation of all primary and secondary lymphoid organs.^4,5^ However, as both the fields of immunology and neuroscience have accumulating and sometimes vast amounts of scientific literature, interrelations between the fields could easily be overlooked. With this “Matters Arising” article, we would like to close this possible gap.

Recently, the group of Moran and Phan published an article in *Nature Communications* about memory B cells that are reactivated in a special microanatomical compartment of lymph nodes.^6^ As this compartment was located directly below the subcapsular sinus, they named it the subcapsular proliferative focus (SPF). The SPF grew out of the clustering and organizing of memory B cells during an induced secondary immune response, and rapid plasma cell generation, proliferation, and antibody secretion into the lymph occurred in this compartment. Almost at the same time, the researchers published a review that put the latter results in a larger context.^7^ Here, they reported that the SPF forms in a larger microenvironment they introduced as a “subcapsular niche”. This subcapsular niche is located all along the lymph node subcapsular region directly below the floor of the subcapsular sinus. The niche is composed of lymphatic endothelial cells that together with interspersed macrophages form the floor of the subcapsular sinus, and marginal reticular cells building the lymph node stroma below the sinus. As these macrophages seem to play a key role in the subcapsular niche microenvironment, they are referred to as subcapsular sinus macrophages (SCS). SCS are specialized for collecting, retaining, presenting, or passing on antigens via their large repertoire of pathogen recognition receptors, thereby influencing innate but also adaptive immune responses. By secreting different cytokine profiles dependent on the antigen recognized, these cells can recruit and activate many innate effector cells present in the subcapsular niche. However, with regard to the focus noted here, in a resting state, they are also the main target of memory B cells and associated follicular memory T cells permanently scanning the SCS for previously recognized antigens. In the case of success, memory B cells together with the associated follicular memory T cells and the SCS rapidly form an SPF for plasma cell generation and antibody production.

These findings lead to the first possible interrelation of the systems: In 2016 and 2018, the group of Tracey and Chavan published two interesting articles about neuronal functions regarding the innervation of the immune system.^8,9^ They showed that sensory neurons can modulate the antigen flow through the lymphatic system and the corresponding draining lymph nodes. If immunized mice were confronted with the same antigen again, the researchers observed a restriction of the antigen flow during the secondary response. As this was a long-lasting phenomenon, the authors suggested an antigen-specific memory leading to the modification of antigen transit through an unknown neuronal circuit. Based on their additional results that neuronal Fc receptors play a role in antigen restriction, the researchers subsequently reported that sensory neurons in the dorsal root ganglia of immunized mice contain antigen-specific antibodies. These neurons could sequester, retain, and release primarily the isotype IgG. Giving evidence for the unknown machinery necessary for IgG synthesis, the authors revealed that the sensory neurons acquire IgG from antibody-secreting plasma cells.

One of many open questions is where these sensory neurons innervate their target structures of the lymphatic system and lymphoid organs, leading to the second possible interrelation of these systems: In 2015 and 2018, our group published articles identifying a new type of innervation in lymphoid organs.^10,11^ Mainly, we described two phenomena. First, we observed countless single immune cells often with antigen-presenting capacity that were partly or totally enclosed by neural structures formed by single neurites reaching these cells. Often, these immune cells were located in the T-cell zone or interfollicular regions of secondary lymphoid organs. Second, we found a dense accumulation of neurites always at the “border” or “gate” sites of all secondary lymphoid organs, which are the main contact or entrance areas for soluble antigen or antigen-presenting cells, respectively. As the many single neurites looked like a network, we named the latter phenomenon the neural nexus. This neural nexus could be detected below the subcapsular sinus in lymph nodes, below the subepithelial dome of Peyer’s patches, below the marginal sinus of the spleen white pulp, and at the outer edges of BALT and NALT.

**Fig. 1:**
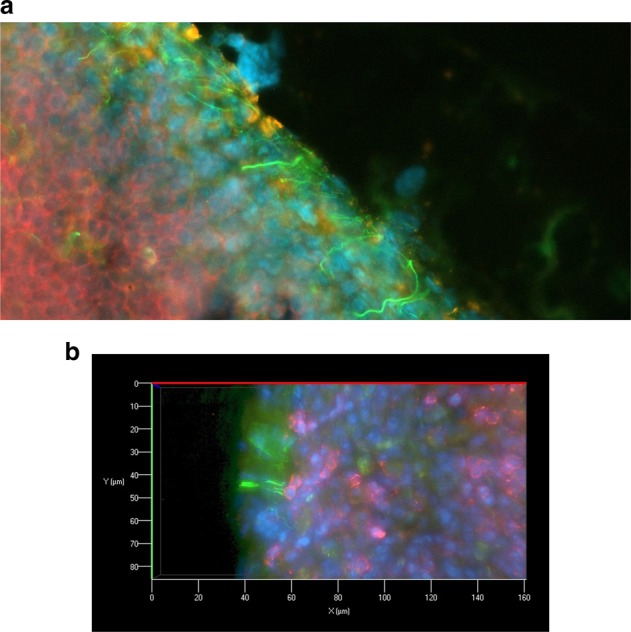
Innervation of the subcapsular niche. **a** The region just below the subcapsular sinus in a lymph node can be seen with CD169-positive subcapsular sinus macrophages (orange) located between the sinus and the outer margin of a follicle with densely packed B cells (red). This region, defined as the subcapsular niche by Moran and Phan, is the same region where our group has always found a dense accumulation of neurites (green), which are in close proximity to the macrophages. **b** Some of the neurites (green) in the subcapsular niche seem to have close contact with B cells (red); therefore, they may acquire IgG from plasma cells, as the Tracey group has shown that isotype in sensory neurons of the dorsal root ganglia. Lymph nodes of C57/BL/6 mice stained with monoclonal anti-neurofilament (green) in panels **a** and **b**, anti-B220 (red) in **a** and **b**, anti-CD169 (orange) in **a** and DAPI (blue)

Furthermore, in the lymph nodes, this neural nexus was located at exactly the same place where the group of Moran and Phan defined the subcapsular niche microenvironment, which may close a gap in our knowledge (see Fig. 1). Therefore, memory B cells also form an SPF in the same area during a secondary immune response generating plasma cells. As these plasma cells differentiate from class-switched memory B cells, they will produce IgG, which is the isotype that Tracey and Chavan detected in sensory neurons of the dorsal root ganglia.

These findings can be pure chance, instead of being related, but the latter results show interesting aspects: neural memory is formed by engrams built by the same neuronal ensembles working on the original sensory input and mediating the reflex pathway.^3,12^ Immunological memory is formed by clones of the same cells responding to the primary antigenic challenge.^13^ In lymph nodes, these memory immune cells have been shown to be located at a place of dense innervation, but this innervation morphology is located in all secondary lymphoid organs at similar antigen entrance sites.^6,11^

Only small populations of memory B and T cells are sufficient to ensure a rapid secondary immune response. Moreover, the time period for the response is independent of where subsequent antigen encounters occur even in large organisms. Therefore, is it possible that the nervous system is involved in orchestrating this response? We strongly suggest further analyses of possible causal interrelations.
